# How Situational Context Impacts Empathic Responses and Brain Activation Patterns

**DOI:** 10.3389/fnbeh.2017.00165

**Published:** 2017-09-04

**Authors:** Yawei Cheng, Chenyi Chen, Jean Decety

**Affiliations:** ^1^Institute of Neuroscience, National Yang-Ming University Taipei, Taiwan; ^2^Department of Physical Medicine and Rehabilitation, National Yang-Ming University Hospital Yilan, Taiwan; ^3^Department of Psychology and Department of Psychiatry and Behavioral Neuroscience, University of Chicago Chicago, IL, United States

**Keywords:** clinical empathy, emotional regulation, functional MRI, situational context, burnout

## Abstract

Clinical empathy, which is defined as the ability to understand the patient’s experience and feelings from the patient’s perspective, is acknowledged to be an important aspect of quality healthcare. However, how work experience modulates the empathic responses and brain activation patterns in medical professions remains elusive. This fMRI study recruited one hundred female nurses, who varied the length of work experience, and examined how their neural response, functional connectivity, and subjective evaluations of valence and arousal to perceiving another individual in physical pain are modulated by the situational context in which they occur (i.e., in a hospital or at home). Participants with longer hospital terms evaluated pain as less negative in valence and arousal when occurring in a hospital context, but not in a home context. Physical pain perceived in a hospital compared to a home context produced stronger activity in the right temporoparietal junction (rTPJ). The reverse comparison resulted in an increased activity in the insula and anterior midcingulate cortex (aMCC). Mediation analysis indicated that reduced personal accomplishment, a symptom of burnout, breaks down the mediation effect of the putamen on context-dependent valence ratings. Overall, the study demonstrates how situational contexts significantly influence individuals’ empathic processing, and that perceiving reward from patient care protects them from burnout.

**Highlights**

- Differences in behavior ratings and brain activations between medical practitioners perceiving others’ pain in a hospital and at home.
- Situational contexts significantly influence individual’s empathic processing.
- Perceiving rewards from patient care protects medical practitioners from burnout.
- Empathy is a flexible phenomenon.

## Introduction

Empathy, the ability to recognize and share the feelings of others, appears to be a situated cognitive process, embedded with specific contextual cues that trigger different automatic and controlled responses (Decety and Jackson, 2004; Melloni et al., 2014). While empathy clearly has a positive role in social interaction, excessive levels of emotional sensitivity may lead to negative outcomes such as personal distress, burnout or desensitization. One apt example is clinical empathy. In the context of caregiving environments, medical practitioners, such as physicians, emergency workers and nurses, have no choice but to deal with people who are suffering or traumatized as part of their everyday routine. How these practitioners respond to, and experience this suffering can have a significant impact on their own well-being (Figley, 2012; Gleichgerrcht and Decety, 2012; Halpern, 2012). Yet, their responses to others’ suffering may be situation-dependent, and drastically vary across social contexts. This view aligns with the second-person approach to social cognition, which suggests that interpersonal understanding is primarily a matter of social interaction and emotional engagement with others (Baez and Ibanez, 2014; Schilbach, 2014).

Clinical empathy, which is defined as the ability to understand the patient’s experience and feelings and view the world from the patient’s perspective, is acknowledged to be an important aspect of quality healthcare. An empathetic attitude is associated with improved patient satisfaction, increased adherence to treatment and a positive influence on patients’ health (Halpern, 2012). But empathy can also be costly. Medical practitioners are frequently exposed to pain and emotional suffering. Such painful reality may take its toll and lead to compassion fatigue, burnout and professional distress. This can result in a low sense of accomplishment and severe emotional exhaustion (Figley, 2012; Gleichgerrcht and Decety, 2012). Physicians who burn out are more likely to report making medical errors, score lower on instruments measuring empathy, make plans to retire early and experience higher job dissatisfaction. These factors have been linked to a decrease in both patient satisfactions with medical care and patient adherence to treatment plans (Dyrbye and Shanafelt, 2011). Evidence about the interaction between empathy and well-being in medical practitioners seems to be mixed (Zenasni et al., 2012). Some studies, based on self-reported questionnaires, suggest that higher levels of dispositional empathy can be associated with compassion fatigue, exhaustion and burnout (Figley, 1995; Nielsen and Tulinius, 2009). Other studies indicate that empathy protects medical practitioners from burnout (Halpern, 2003). For example, medical practitioners with higher dispositional empathy reported more distress in response to stimuli depicting facial expressions of pain (Gleichgerrcht and Decety, 2014) and more emotional exhaustion, a symptom of burnout (Tei et al., 2014). Therefore, it is no surprise that some authors claim that empathy is always beneficial in medical care and allows physicians to complete clinical tasks more accurately while others argue that keeping an emotional distance from patients maintains clinical neutrality (Riess, 2015).

Neuroscience research can help elucidate the relations between interpersonal sensitivity, empathy, situational contexts and caregiving, and ultimately help physicians to maintain high levels of empathy in clinical practice (Decety et al., 2014). One study reported that higher level of emotional exhaustion in nurses is associated with lower activation in the insula and right temporoparietal junction (rTPJ; Tei et al., 2014), a region that plays an important role in self-other distinction and the sense of agency (Silani et al., 2013). Physicians who report higher perspective-taking abilities derive higher satisfaction from perceived treatment success. These physicians showed stronger activation in the rostral part of the anterior cingulate cortex (ACC), a region associated with reward processing, when viewing patient-physician interactions during which the patient was experiencing pain (Jensen et al., 2014).

Another important issue in clinical empathy is that repeated exposure to patients’ suffering may lead to desensitization, which in turn may hamper medical practitioners’ motivation to detect the pain of another person (Franck and Bruce, 2009). Neuroimaging research has reliably documented that perceiving other people in physical or emotional pain elicits neurohemodynamic responses in a specific brain network, including the anterior insula, anterior midcingulate cortex (aMCC), somatosensory cortex (SI/II) and brainstem (Jackson et al., 2006; Lamm et al., 2011). Because these areas belong to the so-called “pain-matrix” (i.e., regions consistently activated by acute physical pain), this overlap between the perception of pain in others and first-hand experience of pain has been interpreted as a neural mechanism by which one may share the pain of another (Decety, 2011; Chen et al., 2012). Activation in that network is modulated by interpersonal relationships, group preferences and attitudes (Decety et al., 2010a; Morrison et al., 2012). However, recent studies challenge this interpretation by showing that activity in the pain-matrix may better be interpreted as increased saliency and relevance to pain-related cues rather than actual empathic processing (Decety, 2015). In support of such an interpretation, prior exposure to stimuli depicting somatic pain did not increase the neural response in the pain matrix, but instead led to decreased activities in these regions (Preis et al., 2013).

Some studies have also reported that medical practitioners are less accurate in identifying painful expressions (Xavier Balda et al., 2000; Kappesser and Williams, 2002). One previous neuroimaging study examined neurohemodynamic responses in physicians and non-physician controls when they viewed short video clips depicting hands and feet being pricked by a needle (painful situations) or being touched by a Q-tip (non-painful situations; Cheng et al., 2007). An increased activation in the pain network was detected in the non-physicians when they attended to the painful situations relative to the non-painful ones. A strikingly different pattern was found in the physicians. The brain regions underpinning executive function, self-regulation (dorsolateral and medial prefrontal cortex, dlPFC/dmPFC) and attention (superior parietal cortex and rTPJ) was activated, rather than the pain network. A follow-up study recorded event-related potentials in physicians when they were presented with the same visual stimuli (Decety et al., 2010b). The results showed an early N110 and a late P3 in the control participants. In contrast, no such early and late ERP responses were detected in the physicians, which indicates that affect regulation has very early effects, inhibiting the bottom-up processing that may lead to negative arousal arising from the perception of painful stimuli.

The desensitization or reduced arousal to others’ pain could be a double-edged sword to medical professions. On the one hand, it could help clinical practitioners to minimize personal distress, combing with elevated brain activities in the regions underpinning executive function, self-regulation and attention, while facing the enormous pain that was expressed by the patients, to provide treatment appropriately. On the other hand, it might also hamper medical practitioners’ motivation to detect the pain of another person.

It remains elusive from the existing neuroscience literatures whether and how work experience impacts changes in behavioral and brain responses to the pain of others in medical practitioners. Furthermore, no study has yet addressed the role of situational context in modulation of these responses in this population. Based on a second-person approach to social cognition, we hypothesized that work experience would modulate the neuro-hemodynamic response to perceiving others’ pain in nurses only within specific situational context, namely the hospital, where the medical treatment was routinely given. Instead of generalizing the desensitized effect of others’ pain to the context outside of work place, the patient healthcare experience should not overall hamper nurses’ motivation to detect the pain of another person.

To gain a better understanding of the interaction between work experience, situational context and well-being in medical practitioners, this study employed a well-validated fMRI empathy paradigm (Cheng et al., 2010; Decety et al., 2013) with one hundred nurses to examine how the pattern of brain activation and subjective evaluations of valence and arousal to perceiving another person in physical pain are modulated by the situational context in which they occur (i.e., in a hospital or at home). Given sexual dimorphism of empathy (Yang et al., 2009; Dehning et al., 2012), only female nurses were enrolled. Given that mediation analysis can be used to investigate the role of intermediate variables that lie on the causal path between 2 variables, it provides a descent way to identify the brain mediators of behavioral changes by applying data from functional neuroimaging (Atlas et al., 2010; Lindquist, 2012). Here, we conducted mediation analyses to examine the causal relationship between work experience in healthcare, neural underpinning of emotional and cognitive empathy, and the context-specific modulation on subjective evaluations. Furthermore, how burnout or reward levels moderated the relations between the amount of work experience and neuro-empathetic responses were included into analyses (Hasselhorn et al., 2004; Jensen et al., 2014).

## Materials and Methods

### Participants

One hundred female nurses were included in this study. All participants were ethnic Chinese aged between 20 years and 55 years. All nurses graduated from a nursing department of a 4-year medical college or 5-year junior college. Their hospital terms ranged from 0.2 to 29.8 years (*M* = 7.6, SD = 6.3). None of the control participants had any healthcare work experience. This study was carried out in accordance with the Declaration of Helsinki. All subjects gave written informed consent for the study, which was approved by the Ethics Committee of National Yang-Ming University Hospital.

### Stimuli Selection

A total of 60 visual stimuli were used to depict injured or uninjured body parts (hands and feet). The stimuli were selected *ad hoc* for the study from 120 pictures based on the ratings provided by 30 adults (15 females, aged 24 ± 4.5 years), who did not take part in the fMRI study. Using a 5-point bivariate valence scale (Berntson et al., 2011), 30 pictures were selected with the highest valence ratings (4.1 ± 0.8) and 30 with the lowest valence ratings (0.4 ± 0.6) in order to represent the painful and neutral stimuli, respectively. Additionally, 20 pictures were used for priming situational contexts. Ten hospital pictures depicted nurses and doctors working in hospital scenes and 10 pictures were home scene showing family members doing daily activities. The study thus included four categories of stimuli: pain at home context (PHC), neutral at home context (NHC), pain at work context (PWC) and neutral at work context (NWC). For the sake of carefully controlling the validity of the experimental paradigm, we recruited another 25 controls to re-confirm that control participants showed a comparable pattern of neuro-hemodynamic responses along with many existed literatures that were associated with pain empathy (Supplementary Table S1).

### Procedures

One week before fMRI scanning, each participant took part in the assessment of a number of dispositional measures, including the Interpersonal Reactivity Index (IRI; Davis, 1996), Toronto Alexithymia Scale-Taiwan version (TAS-20-T; Bagby et al., 1994; Lin and Chan, 2003) and Maslach Burnout Inventory Human Service Survey (MBI-HSS; Maslach et al., 1996). Burnout is a multidimensional process with three constructs: emotional exhaustion, depersonalization and reduced accomplishment (Maslach and Jackson, 1981).

**Figure 1:**
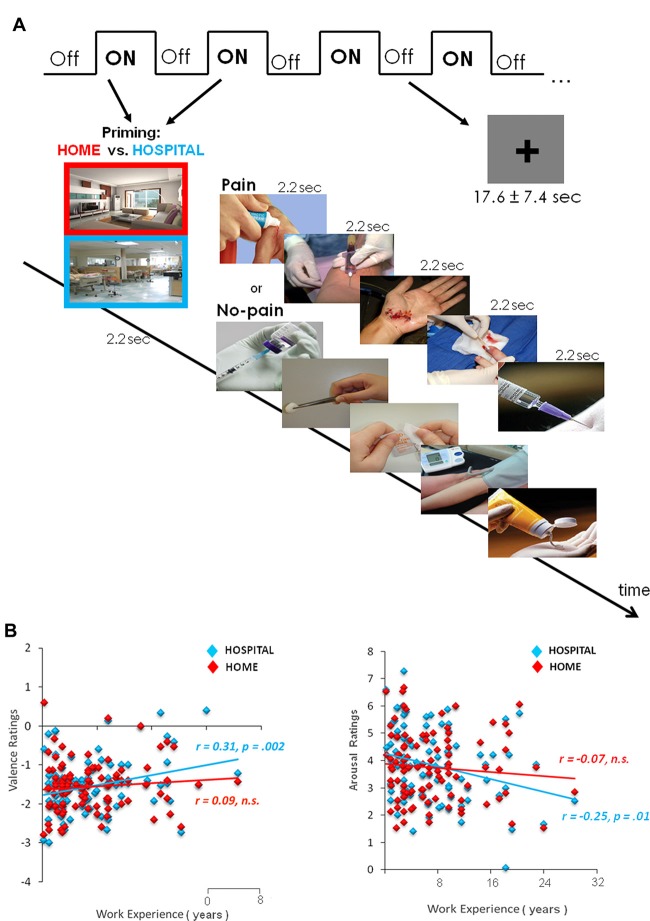
Experimental design and subjective ratings of valence and arousal. **(A)** The fMRI scanning used a mixed block design. Each block was preceded by one photo for priming the situational context (Home or Hospital). After scanning, participants rated the stimuli in both valence and arousal. **(B)** Context-dependent valence and arousal ratings of the pain stimuli. Within the hospital context, nurses who had longer hospital terms (years) tended to evaluate the pain stimuli less negatively in both valence and arousal. Fisher r-to-z transformation confirms that the hospital context significantly differed correlation coefficients from the home context (Δ*z* = 2.15, *p* < 0.05).

The fMRI used a mixed block design (13.2 s ON/17.6 ± 7.4 s OFF; Figure 1A). There were a total of two runs. Each run consisted of eight ON intermixed with eight OFF blocks. Each ON block consisted of one photo (2200 ms) for priming and five trials (2200 ms each) from painful or neutral stimuli. To elicit the priming effect of situational context on pain empathy, we showed the picture cue participants about which context to employ at the beginning of each block (at home or in a hospital) and gave the instructions, including the home context (“Imagine that you are facing one person, for example, a friend or family member, in the pain/no-pain at home”) and hospital context (“Imagine that you are facing the pain/no-pain with the same person in the hospital”). The OFF condition remaining on screen for 17.6 s was followed by a jittered fixation cross (5.0 ± 4.7 s) presented against a gray background in order to avoid the expectation and multicollinearity among regressors. The order of the stimulus condition was randomized and counterbalanced across runs.

After fMRI scanning, participants were asked to rate the stimuli for their valence and arousal. Participants positioned a cursor on a bivariate display (a 5 × 5 grid) in which the horizontal dimension indicated the amount of positive valence and the vertical dimension indicated the amount of negative valence toward these stimuli (Norris et al., 2010). For arousal ratings, participants positioned the cursor on a single dimension display (9-point scale).

### Functional MRI Acquisition and Data Analysis

Images were acquired using a 3T Siemens Magnetom Trio-Tim magnet. For functional images, 3-mm-thick transverse slices oriented along the AC-PC line were continuously collected using an echo-planar imaging sequence (TR/RE = 2200/30 ms, FOV = 220 mm, flip angle = 90°, matrix = 64 × 64). Functional images were co-registered to the T1 template, and a skull-stripped image was created from the segmented gray matter, white matter and CSF images. These segmented images were then combined to create a subject-specific brain template. EPI images were realigned and filtered (128 s cutoff), then co-registered to these brain templates, normalized to MNI space and smoothed (8 mm FWHM).

Functional MRI data were processed with SPM8 (Wellcome Department of Imaging Neuroscience, London, UK) in MATLAB 7.0 (MathWorks Inc., Sherborn, MA, USA). Data were entered into a general linear model, with movement parameters as nuisance regressors. A two-stage general linear model was used to examine the effect sizes of each condition. At the first level analysis, four conditions (PHC, NHC, PWC, NWC) were modeled separately with a duration of 13.2 s beginning at the onset of the priming photo. The null event (fixation) was modeled with the duration 17.6 ± 7.4 s. Linear contrasts were applied to obtain parameter estimates. At the second-level analysis, images of parameter estimates from the first level were collapsed into a 2 × 2 factorial design with stimulus (Pain vs. Neutral) and context (Work vs. Home) as the within-subject variables. Groupwise effects for the following contrasts were corrected for multiple comparisons family-wise error rate (FWE) of *p* < 0.05 (cluster size *k* > 20). To explore the extent to which context-dependent hemodynamic changes found in the group-wise analysis were related to subjective evaluations, whole brain correlation analyses were conducted with valence and arousal ratings in the home or hospital context, respectively (thresholded at *p* < 0.001, and cluster extent of at least 20 contiguous voxels, FWE at *p* < 0.05).

In addition, valence and arousal ratings were entered as covariates and thresholded at an uncorrected *p* < 0.001 with a cluster extent of at least 25 contiguous voxels (FWE of *p* < 0.05). The MarsBar toolbox was used to extract the mean activity within a 6-mm-radius sphere (FWE-corrected, *p* < 0.05) from pre-defined regions of interest (ROIs). The ROI coordinates (rTPJ: 62, −54, 16; right insula: 44, −4, 0; aMCC: 6, 18, 30 in Montreal Neurological Institute (MNI)) were selected from fMRI meta-analyses of theory of mind (ToM) and empathy (Lamm et al., 2011; Bzdok et al., 2013, 2015). The mean activity of each ROI was subjected to repeated measures ANOVA with stimulus (Pain vs. Neutral) and situational context (Work vs. Home) as the within-subject variables.

### Functional Connectivity Analysis

The cluster in the insula as a result of the second level analysis ((PHC − NHC) − (PWC − NWC)) was selected as a seed given this polysensory region being an integrative hub of the salience network (Menon and Uddin, 2010). Psychophysiological interaction (PPI) was used to investigate task-specific changes in the relationship between hemodynamic responses in different brain areas. Technically, the time series of the first eigenvariates of the BOLD signal were temporally filtered, mean corrected and deconvolved to generate the time series of the neuronal signal for the source region—the insula—as the physiological variable in the PPI. The psychological variable represented the time course of the contrast between painful and non-painful trials. The interaction term in the resulting SPM showed areas with selective connectivity to the insula across the psychological contrast of Pain vs. Neutral. The PPI analysis was performed for each participant, and the resulting images of contrast estimates were entered into a random-effects group analysis. PPI analyses were separately run for hospital (PWC − NWC) and home (PHC − NHC) contexts to acquire the functional connectivity seeded in the right insula (*x* 44, *y* −4, *z* 0) between different situational contexts. Results on second-level analysis were reported at a voxel-wise statistical cutoff of *p* < 0.001 and a spatial extent threshold of *k* > 25 voxels.

### Mediation Analysis

Mediation Effect Parametric Mapping was used to test specific hypotheses about brain-behavior relationships (Baron and Kenny, 1986; Wager et al., 2008). Based on the conceptual framework of a mediation effect (MacKinnon et al., 2007), work experience (hospital terms in years) was selected as the predictor, the context-dependent valence or arousal ratings as the outcome, and the neuro-hemodynamic activity in each ROI as the mediator.

Mediation analyses were conducted in a sequential series of steps. Step 1 identified the context-dependent valence and arousal ratings to pain stimuli. Step 2 used the whole brain correlation analyses to identify the ROIs involved in neuro-empathetic reactions. Step 3 tested whether each ROI mediated the linkage between work experience and context-dependent valence and arousal ratings. Step 4 tested how burnout levels, as indicated by the scores on each MBI-HSS subscale, moderated these existing brain-behavior relations.

Path *a* coded the link in which the predictor variable must be related to the mediator. Path *b* coded the link in which the mediator must be directly related to the outcome, controlling for work experience. The mediation effect (a*b) must be significant, which amounts to a statistical test on the product of the *a* and *b* path coefficients. Equivalently, the test for the predictor-outcome relationship would be significantly reduced by the inclusion of the mediator in the path model. We refer the overall predictor-outcome relationship as the *c* effect, and control the direct effect for the mediator as *c*′. The a*b effect was to test the significance of *c* − *c′*.

## Results

### Dispositional Measures and Subjective Ratings

Scores on the depersonalization (*r* = −0.21, *p* = 0.039) and personal accomplishment subscales (*r* = 0.23, *p* = 0.021) of the MBI-HSS were associated with work experience (years). However, no other dispositional measures showed any correlation.

Subjective valence and arousal ratings of pain stimuli in a hospital context were related to years of work experience (Figure 1B). Nurses with more work experience evaluated pain stimuli as less negative in valence (*r* = 0.31, *p* = 0.002) and arousal (*r* = −0.25, *p* = 0.01). In contrast, in a home context, work experience was not related to valence (*r* = 0.09, *p* > 0.1) and arousal (*r* = −0.07, *p* > 0.1). Fisher *r*-to-*z* transformation Δ*z* = 2.15, *p* < 0.05, confirmed significantly different correlation coefficients between contexts. Direct comparisons between home and hospital contexts on valence (*t*_(99)_ = 0.045, *p* = 0.96) and arousal (*t*_(99)_ = −1.04, *p* = 0.03) ratings did not reach significance.

### fMRI Group-Wise Comparisons

For the pain effect ((PHC + PWC) − (NHC + NWC)), there was significant increment in hemodynamic activity in the brain regions involved in pain matrix, including the anterior insula, aMCC, supplementary motor area, inferior frontal gyrus, dlPFC/dmPFC, SI/II and periaqueductal gray (Table 1).

**Table 1:** Pooled group results for all participants (*N* = 100).

Brain region	MNI coordinates			Peak *T*
	*x*	*y*	*z*	
**PAIN > NO-PAIN**				
- Periaqueductal gray	−6	−26	−6	4.62
- Anterior mid-cingulate cortex	2	22	40	4.98
- Dorsomedial prefrontal cortex	0	56	30	4.92
R Anterior insula	32	16	0	5.08
R Inferior frontal gyrus	46	8	26	8.14
R Dorsolateral prefrontal cortex	48	50	10	5.83
R Supplementary motor area	12	12	72	6.11
R Putamen	30	−22	−2	4.9
R Supramarginal gyrus	66	−18	32	4.99
R Posterior cingulate cortex	8	−38	24	4.1
L Anterior insula	−40	20	2	4.77
L Inferior frontal gyrus (pars opercularis)	−42	6	24	6.51
L Supplementary motor area	−10	18	68	6.44
L Inferior frontal gyrus	−38	34	12	5.83
L Thalamus	−16	−10	8	5.41
L Dorsolateral prefrontal cortex	−20	68	14	5.03
L Precuneus	−22	−56	42	4.92
L Postcentral gyrus	−58	−20	30	4.88
L Ventrolateral prefrontal cortex	−32	30	−8	4.81
**HOSPITAL > HOME**				
L Dorsolateral prefrontal cortex	−50	−4	36	4.28
L Postcentral gyrus	−40	−18	38	5.52
L Cingulate gyrus	−14	4	42	3.73
L Lingual gyrus	−26	−64	−8	3.43
R Dorsolateral prefrontal cortex	50	−2	38	4.49
R Temporal pole	62	6	2	3.92
R Lingual gyrus	20	−48	−8	3.41
**(PHC − NHC) vs. (PWC − NWC)**				
R Insula	44	−4	0	3.87
R Inferior temporal gyrus	58	−20	−22	3.91
R Putamen	26	18	0	3.44
L Cingulate gyrus	−10	10	30	3.21
R Anterior mid-cingulate cortex	6	18	30	1.86*
**(PWC − NWC) vs. (PHC − NHC)**				
R Temporoparietal junction	64	−54	16	1.75*

*All clusters reached significance at family-wise error rate (FWE)-corrected p < 0.05 (thresholded at p < 0.001, cutoff, t = 3.11, uncorrected, a spatial extent threshold k = 20), except those marked by a star, which were taken from pre-defined regions of interest (ROIs) and significant at uncorrected p < 0.05*.

**Figure 2:**
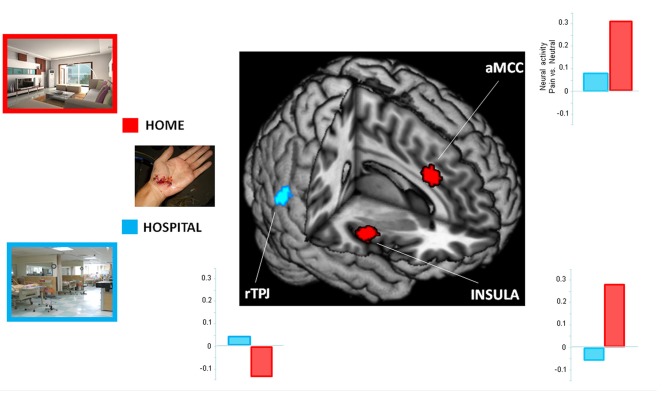
Context-dependent neuro-hemodynamic activity in response to viewing pain stimuli. The regions of interest (ROIs) included the right temporoparietal junction (rTPJ; *x* 62, *y* −54, *z* 16), right insula (*x* 44, *y* −4, *z* 0) and anterior midcingulate cortex (aMCC; *x* 6, *y* 18, *z* 30). The home context had greater hemodynamic activity in the insula and aMCC than did the hospital context. The reverse comparison showed a stronger activity in the rTPJ. Clusters from the whole-brain contrast was thresholded at *p* < 0.001 with an extent threshold of *k* > 25 (FWE < 0.05).

To examine the influence of situational context on pain perception, we performed an interaction analysis. When participants perceived painful stimuli within a hospital relative to a home context ((PWC − NWC) vs. (PHC − NHC)), increased activity was detected in the rTPJ. The reversed comparison ((PHC − NHC) vs. (PWC − NWC)) showed increased activity in the insula and aMCC. Results from the ROI analyses are presented in Figure 2.

### Functional Connectivity

Situational contexts elicited distinct patterns in functional coupling when participants viewed painful stimuli. The hospital context, but not the home context, was associated with a positive coupling between the insula and the MCC and a negative coupling with the ventromedial prefrontal cortex (vmPFC; *p* < 0.001 and *k* > 25 voxels).

### Correlations between Neuro-Hemodynamic Activity and Subjective Ratings

Whole brain correlation analyses showed that, within the home context, valence ratings inversely predicted hemodynamic activity (Pain vs. Neutral) in the insula (46, 6, 2; −44, 4, −4). Within the hospital context, more positive valence ratings predicted stronger activities in the putamen (24, −8, 10) and dlPFC (−42, 18, 30). In addition, arousal ratings inversely predicted activities in the ACC (−14, 32, 28) and dmPFC (−6, 26, 52). These results were thresholded at individual *p* < 0.001, and cluster extent of at least 20 contiguous voxels, FWE at *p* < 0.05. The neuro-hemodynamic estimates in these regions were extracted for mediation analyses.

### Mediation Analysis Results

**Figure 3:**
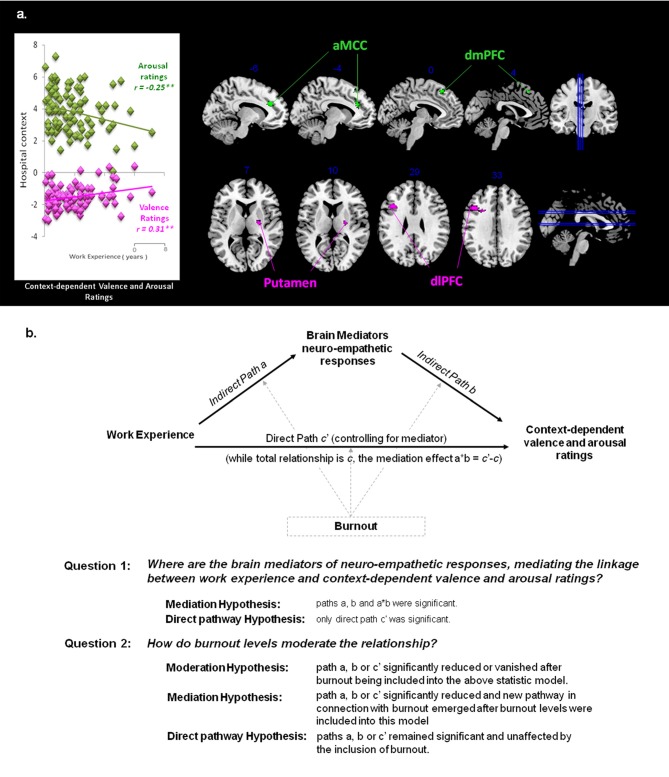
Hypothesis for mediation analysis. **(A)** Context-dependent valence and arousal ratings. The correlation between subjective ratings and work experience was specific to the context. Significant correlations occurred between context-dependent subjective ratings and neuro-empathetic reaction (Pain vs. Neutral). Suprathreshold voxels (*p* < 0.05 corrected for voxel-wise comparisons) are displayed across four coronal and sagittal sections on the ch2bet template, using MRIcron software (http://www.sph.sc.edu/comd/rorden/mricron/). The hemodynamic response in the anterior cingulate cortex (ACC; −14, 32, 28) and dorsomedial prefrontal cortex (dmPFC; −6, 26, 52) was significantly negatively correlated with arousal ratings (shown in green), whereas the response in the putamen (24, −8, 10) and dorsolateral prefrontal cortex (dlPFC; −42, 18, 30) was positively correlated with valence ratings (shown in magenta). These areas are the main ROIsfor the mediation analysis. **(B)** Mediation Model and Hypotheses. While observing the context-dependent valence and arousal ratings, we selected work experience as the predictor variable, and performed analyses to test if the above ROIs were the mediators of the linkage between work experience and subjective ratings of valence and arousal. Whether and how each scale of Burnout moderates the existing model and area of interest are considered as well.

While work experience (hospital terms in years) was selected as the predictor and the context-dependent valence or arousal ratings as the outcome, the neuro-hemodynamic activity in each regions, identified by whole brain correlation analyses, was the mediator (Figure 3A). Driven by the mediation model and hypotheses (Figure 3B), we performed analyses to test if each ROI was the mediators of the linkage between work experience and subjective ratings of valence and arousal. Moderation effects of each scale of Burnout were also examined on areas of interest.

#### Putamen and dlPFC Mediate the Linkage between Work Experience and Context-Dependent Valence Ratings

**Figure 4:**
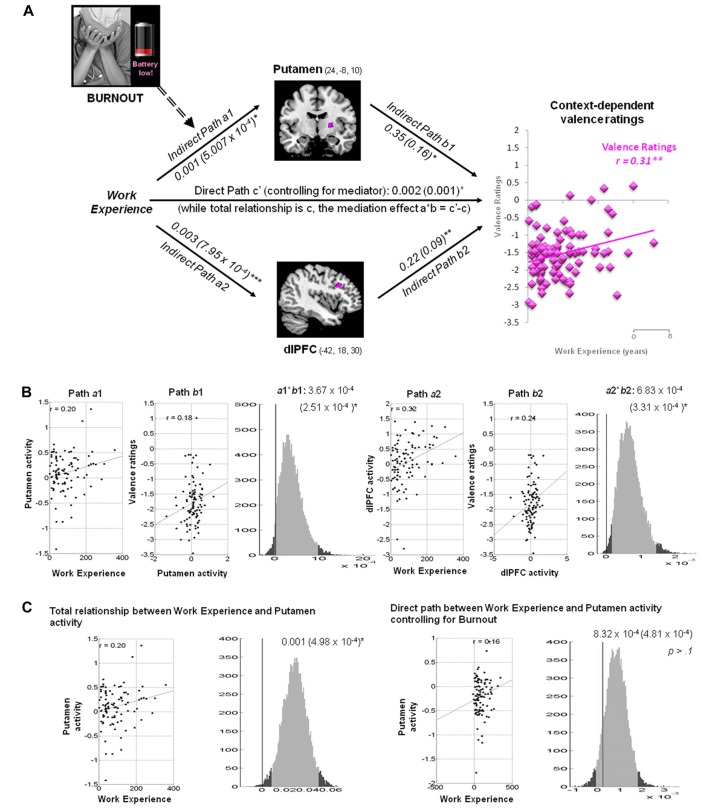
Mediation analysis of context-dependent valence ratings. **(A)** Path diagram demonstrates the relationship between variables in the path model. Work experience (left) as the predictor variable predicts the hemodynamic activity in the putamen (top) and dlPFC (bottom). The connection of work experience to each brain mediator (putamen or dlPFC) is the *a* path. The lines are labeled with path coefficients, and standard errors are shown in parentheses. The connection of each mediator to the outcome (valence ratings) is the *b* path. They are calculated controlling for work experience and for other mediators, as the standard in mediation models. The inclusion of burnout levels into the mediation model abolishes the causal relationship between work experience (left panel), putamen activity (top), and context-dependent valence ratings (right panel). Scores on reduced personal accomplishment, as one of burnout constructs, moderates the mediation effect of putamen (path *a*1, dash arrow). ***p < 0.001, **p < 0.01, *p < 0.05, two-tailed. The direct path is the *c*′ path, which is calculated controlling for both mediators. **(B)** Substantiation of the mediation path *a*, *b* and *c*. Regression scatterplots depict the relationships between predictor (i.e., working experience) and ROIs (path *a*1 and *a*2). Partial regression scatterplots demonstrate the relationships between ROIs (putamen and dlPFC) and valence ratings (path *b*1 and *b*2). The mediation effect (*a*1*b1 and *a*2*b2) is substantiated by the bootstrapped distributions. The range on the *x*-axis spanned by the lighter gray portion of the histogram is the 95% confidence interval for the effect. **(C)** A significant correlation between work experience and putamen (path a1) was found before Burnout being included into the mediation model (left panel). One of the Burnout subscales, reduced personal accomplishment, affected achievement-motivational processing in the putamen and modulated the mediation effect of putamen (path a1), thus abolish the causal relationship between work experience, putamen and valence processing in hospital context (right panel).

Within the hospital context, the hemodynamic activity in the putamen was positively associated with work experience and predicted more positive valence ratings of the pain stimuli: *a* = 0.001, *Z* = 2.37, *b* = 0.35, *Z* = 2.17 and a*b = 0.0004, *Z* = 2.22, all *p* < 0.05 (Figure 4). The activity in the dlPFC was positively associated with work experience, and predicted more positive valence ratings: *a* = 0.003, *Z* = 3.51, *b* = 0.22, *Z* = 2.65 and a*b = 0.0006, *Z* = 2.82, all *p* < 0.05.

#### Burnout Moderates the Mediation Effect of Putamen on Context-Dependent Valence Ratings

Three subscales of MBI-HSS, including emotional exhaustion, depersonalization and reduced personal accomplishment, were included to test if burnout levels affected path *a, b* and a*b. The indirect path *a* between work experience and putamen activity in the valence model (*a* = 0.001, *Z* = 2.41, *p* = 0.016) was diminished after the inclusion of scores on reduced personal accomplishment (*a* = 8.32 × 10^−4^, *Z* = 1.8, *p* > 0.10). The remaining paths were unaffected by other subscales.

#### ACC and dmPFC Mediate the Linkage between Work Experience and Context-Dependent Arousal Ratings

**Figure 5:**
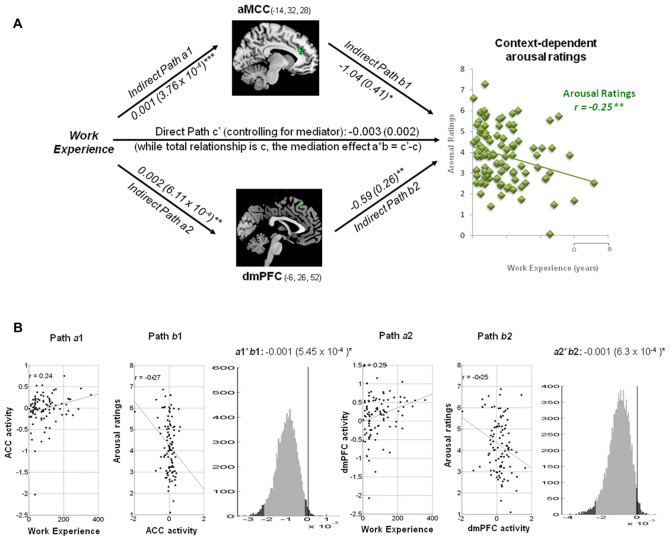
Mediation analysis on context-dependent arousal ratings. **(A)** Path diagram shows the relationship between variables in the path model. Work experience (left) as the predictor variable predicts the activity in the ACC (top) and dmPFC (bottom). The connection of work experience to each brain mediator (ACC or dmPFC) is the *a* path. The lines are labeled with path coefficients, and standard errors are shown in parentheses. The connection of each mediator to the outcome (arousal ratings) is the *b* path. They are calculated controlling for work experience and for other mediators, as the standard in mediation models. ***p < 0.001, **p < 0.01, *p < 0.05, two-tailed. The direct path is the *c*′ path, which is calculated controlling for both mediators. **(B)** Substantiation of the mediation path *a*, *b* and *c*. Regression scatterplots demonstrate the relationships between predictor (work experience) and ROIs (path *a*1 and *a*2). Partial regression scatterplots depict the relationships between ROIs (ACC and dmPFC) and arousal ratings (path *b*1 and *b*2). The mediation effect (*a*1*b1 and *a*2*b2) is substantiated by the bootstrapped distributions. The range on the *x*-axis spanned by the lighter gray portion of the histogram is the 95% confidence interval for the effect. None of each Burnout subscale modulated the mediation effect of arousal ratings.

Within a hospital context, the hemodynamic activity in the ACC was positively associated with work experience, but predicted less arousal ratings of pain stimuli: *a* = 0.001, *Z* = 3.45, *b* = −1.04, *Z* = −2.36 and a*b = −0.001, *Z* = −2.09, all *p* < 0.05 (Figure 5). The activity in the dmPFC was positively associated with work experience, but predicted less arousal ratings of the pain stimuli: *a* = 0.002, *Z* = 2.99, *b* = −0.59, *Z* = −2.30 and a*b = −0.001, *Z* = −1.96, all *p* < 0.05.

## Discussion

Medical practitioners regularly perceive and react to others’ suffering, but this can be costly to their well-being. It is unclear how changes in medical practitioners’ behavioral and brain responses when viewing others in pain are influenced by their work experience and situational context.

In spite of the close association between work experience and age (*r* = 0.95), the context-dependent subjective ratings of the painful stimuli cannot be explained by the aging process itself. One previous study using similar stimuli did not find any age-related changes in subjective ratings in individuals between 20 years old and 80 years old (Chen et al., 2014). Measures of dispositional empathy throughout the adult lifespan showed an inverse-U-shaped pattern across age (O’Brien et al., 2013). In the present study, nurses’ work experience (in year) had a linear effect on the subjective ratings and burnout levels. Medical practitioners who have burnout might tend to change their career path.

Interestingly, nurses with longer work experience evaluated pain stimuli in a hospital context as less negative in both valence and arousal. They also reported less depersonalization and better personal work accomplishment compared with less experience nurses. Experienced nurses may have higher quality and more cost-effective care (Bartel et al., 2014). Context-dependent subjective ratings in relation to work experience may reflect achievement-related processes that modulate motivation to empathy-eliciting stimuli (Takeuchi et al., 2014).

Consistent with previous fMRI studies of pain empathy (Lamm et al., 2011 for a meta-analysis), significant hemodynamic activity was detected in the pain matrix when the nurses perceived pain stimuli (see Table 1). In addition, 25 participant controls included in this study also showed a similar pattern of neuro-hemodynamic responses (Supplementary Table S1). One previous study showed that the anterior insula and aMCC were significantly activated in the controls, but not in the physicians, who instead showed activation of the rTPJ (Cheng et al., 2007). The present study extends these results by demonstrating that the home context led to a stronger activation in the insula and aMCC than did the hospital context, whereas the reverse comparison showed increased activation in the rTPJ.

In a hospital context, rather than a home context, the insula elicited by pain stimuli revealed a negative functional connectivity with the vmPFC, and a positive coupling with the MCC. A neuroimaging study with physicians found a negative covariation of the mPFC with the insula in response to viewing patients being pricked by needles (Cheng et al., 2007). The negative connectivity of the insula with the mPFC elicited by painful stimuli could reflect a cognitive inhibition of their affective processing to patient’s distress, as the mPFC is involved in ToM and emotion regulation (Cheng et al., 2010; Etkin et al., 2011; Zaki and Ochsner, 2012). This finding is consistent with a detached perspective adopted by some medical practitioners. The detachment, orchestrated by executive function, blunts potential emotional contagion from patients’ suffering and allows medical practitioners to respond to patients with adequate assistance.

Importantly, the two situational contexts produced distinct associations between the subjective ratings of valence and the neurohemodynamic response to perceiving painful stimuli. The negative valence ratings of pain in a home context were positively coupled with enhanced activity in the insula. The hospital context, however, led to more positive valence ratings and predicted increased activity in the putamen and dlPFC. It can be argued that medical practitioners must not become detached and callous except when working in a hospital. The neuro-hemodynamic response in the insula, a region that plays a key role in empathy by mapping internal states of bodily and subjective feelings, suggests that medical practitioners are more emotionally engaged when the pain is perceived in the home context (Banissy et al., 2012). On the other hand, the putamen, as part of the striatum, is involved in the processing of reward (O’Doherty et al., 2002; Mizuno et al., 2008). The dlPFC is implicated in executive control of negative emotions (Ochsner et al., 2004) and emotion regulation of empathy (Decety, 2011). Healthcare involves neural representations of treatment expectation, reward and empathy (Jensen et al., 2014). The response in the putamen and dlPFC reflects how medical training and expertise enables medical practitioners to dissociate from vicariously sharing patients’ suffering and thus from becoming overwhelmed by negative emotions. Furthermore, in addition to situational context, interpersonal variables, implicit attitudes and group preferences could either strengthen or weaken the neural network implicated in empathy for pain (Decety and Cowell, 2014). Here, to probe the effect specific to situational context, participants were instructed to imagine to face the same person (for example, a friend or family member) who was in the pain/no-pain in both of home and hospital contexts.

Mediation analyses indicate that reduced personal accomplishments, one aspect of burnout, moderates the mediation effect of putamen activity on context-dependent valence ratings. Burnout as a multidimensional process includes emotional exhaustion, depersonalization and reduced personal accomplishment (Maslach and Jackson, 1981). Nurses with higher dispositional empathy were more prone to emotional exhaustion, which was associated with lower activation in the insula and rTPJ (Tei et al., 2014). Our findings also demonstrate that nurses with more burnout symptoms were less likely to have longer work experience and to give positive valence ratings. Nurses with more burnout symptoms also showed less achievement- related putamen activation when viewing pain in a hospital context. These findings support the notion that perceiving reward from patient care can protect medical practitioners from burnout (Basińska and Wilczek-Ruzyczka, 2013).

Some limitations of this study must be acknowledged. First, co-effects of expectations of the nurses with respect to hospital and home contexts might contribute to contextual effect for empathic neural responses. Second, the studies on occupational wellbeing in nurses have shown significant associations between coping strategies, personality characteristics, burnout and clinical performance (Adriaenssens et al., 2015; Cañadas-De la Fuente et al., 2015). High neuroticism with the opposite of emotional stability tends to use non-effective coping strategies, such as, avoidance and distraction, and can lead to higher levels of burnout. Hence, future studies to address the interplay between working experience, coping strategies and neural underpinning of pain empathy are warranted.

## Conclusion

Contextual modulation of empathy represents an adaptive advantage, making behavior more sensitive to different environment conditions (Melloni et al., 2014). A better understanding of how situational context influences the neuro-cognitive mechanisms underpinning emotional sensitivity and empathy in medicine can contribute to preventing serious health hazards and risks in medical professionals. It also sheds light on the challenge to achieve an appropriate balance between empathy and clinical distance. Detachment is often seen as necessary not only to avoid burning out or losing control, as well as allow practitioners to provide objective medical care. Our results showed that, instead of generalization of desensitizing effect of others’ pain and suffering outside of the workplace, healthcare experience did not hamper nurses’ motivation to detect the pain of another person at home context. Working experience with less depersonalization and better personal work accomplishment could shape the brain of medical professions in the way to offer healthcare benefits to both caregivers and patients. Switching perspectives between hospital and home contexts might help medical professions release from workplace stress. Of note, perceiving rewards from patient care protects medical practitioners from burnout.

## Author Contributions

YC and JD conceived and designed the experiments, wrote the first draft of the manuscript. CC and YC collected the data, contributed to data analysis. All authors contributed to data interpretation and manuscript write-up.

## Conflict of Interest Statement

The authors declare that the research was conducted in the absence of any commercial or financial relationships that could be construed as a potential conflict of interest.
