# Supplementary material for: How Situational Context Impacts Empathic Responses and Brain Activation Patterns
Source: Front Behav Neurosci. 2017 Sep 4;11:165. doi: 10.3389/fnbeh.2017.00165 (PMC5591329; doi:10.3389/fnbeh.2017.00165)
Supplement: Supplementary file 1 [file Table_1.doc]

**SUPPLEMENTARY MATERIALS**

**Table s1: Results for the naive controls (*n* = 25)**

|  | MNI coordinates | | |  | Cluster size (mm^3^) |
| --- | --- | --- | --- | --- | --- |
| Brain region | x | y | z | *Z* score | *P* < 0.001 |
| ***Pain effect [(MP+HP)-(MNP+HNP)]*** | | | | | |
| L Cuneus | -7 | -85 | 21 | 5.28 | 572 |
| L Declive | -7 | -78 | -18 | 4.63 | 572 |
| L Precuneus | -14 | -80 | 51 | 5.25 | 69 |
| L Superior parietal lobule | -7 | -75 | 60 | 3.97 | 69 |
| L Superior frontal gyrus | -17 | 7 | 69 | 4.89 | 49 |
| L Cerebellum | -34 | -51 | -27 | 4.60 | 39 |
| L Lentiform nucleus | -17 | 0 | 0 | 4.55 | 111 |
| L Caudate nucleus | -14 | -10 | 18 | 3.73 | 111 |
| L Inferior frontal gyrus | -58 | 14 | 6 | 4.52 | 352 |
| L Insula | -44 | 10 | 3 | 4.49 | 352 |
| R Inferior frontal gyrus | 37 | 27 | 3 | 4.49 | 178 |
| R Superior frontal gyrus | 2 | 20 | 51 | 4.13 | 107 |
| L Anterior cingulate | -10 | 24 | 36 | 3.65 | 107 |
| L Middle frontal gyrus | -31 | 58 | 21 | 3.84 | 24 |
| L Precentral gyrus | -48 | -10 | 54 | 3.83 | 40 |
| R Precentral gyrus | 31 | 7 | 36 | 3.62 | 23 |
| L Inferior partial lobule | -54 | -44 | 57 | 3.60 | 12 |
| ***Context effect [(MP+MNP)-(HP+HNP)]*** | | | | | |
| None |  |  |  |  |  |
| ***Context effect [(HP+HNP)-(MP+HMP)]*** | | | | | |
| None |  |  |  |  |  |
| ***Interaction effect [(MP-MNP)-(HP-HNP)]*** | | | | | |
| None |  |  |  |  |  |
| ***Interaction effect [(MP-MNP)-(HP-HNP)]*** | | | | | |
| None |  |  |  |  |  |
